# Efficacy and safety of different bupivacaine concentrations in periarticular infiltration combined with adductor canal block for bilateral total knee arthroplasty: a randomized controlled trial

**DOI:** 10.1186/s43019-024-00211-y

**Published:** 2024-01-19

**Authors:** Sukanya Dej-arkom, Pawinee Pangthipampai, Weerawadee Chandranipapongse, Somruedee Chatsirichareonkul, Rapeepat Narkbunnam, Keerati Charoencholvanich, Suwida Tangchittam, Arissara Iamaroon

**Affiliations:** 1 Department of Anesthesiology, Faculty of Medicine Siriraj Hospital, Mahidol University, Bangkok, 10700 Thailand; 2 Department of Pharmacology, Faculty of Medicine Siriraj Hospital, Mahidol University, Bangkok, 10700 Thailand; 3 Department of Orthopedic Surgery, Faculty of Medicine Siriraj Hospital, Mahidol University, Bangkok, 10700 Thailand

**Keywords:** Adductor canal block, Pain management, Periarticular infiltration, Plasma bupivacaine concentrations, Simultaneous bilateral total knee arthroplasty

## Abstract

**Background:**

Pain management for bilateral total knee arthroplasty (BTKA) often combines adductor canal block (ACB) with periarticular infiltration (PAI). However, concerns arise regarding local anesthetic toxicity. This study evaluated the efficacy and safety of different bupivacaine concentrations in simultaneous BTKA.

**Methods:**

Patients undergoing simultaneous BTKA under spinal anesthesia were included in the study. They received ACB with 50 mg bupivacaine for each thigh. The patients were then randomized into two groups. Group A was administered a PAI of 100 mg bupivacaine per knee (totaling 300 mg bupivacaine for ACB and PAI). Group B received a PAI with 50 mg bupivacaine per knee (totaling 200 mg bupivacaine for ACB and PAI). Postoperative pain was assessed using a visual analog scale at 4-h intervals for 48 h after surgery. Plasma bupivacaine concentrations were measured at eight specified times. Postsurgery walking ability was also evaluated.

**Results:**

Among the 57 participants analyzed, visual analog scale pain scores revealed no significant differences between the two groups. An interim analysis of plasma bupivacaine concentrations in both groups indicated no significant disparities. In group B, 93.1% managed to walk with assistance within 48 h, as opposed to group A’s 71.4% (*P* = 0.041).

**Conclusions:**

Combining ACB with 100 mg bupivacaine and PAI with another 100 mg bupivacaine provided effective pain relief. This combination also had a better safety profile and led to more patients walking postsurgery than when combining ACB with 100 mg bupivacaine and PAI with 200 mg bupivacaine. Thus, ACB combined with PAI with a total dose of 200 mg bupivacaine appears suitable for simultaneous BTKA.

*Trial registration*: ClinicalTrials.gov (NCT03249662).

## Introduction

Postoperative pain is a common concern for patients undergoing total knee arthroplasty (TKA), with more than 50% reporting moderate to severe pain after surgery [1, 2]. Studies have shown that bilateral TKA (BTKA) results in higher levels of pain [3, 4] and opioid consumption than unilateral TKA [4]. To address this, the literature suggests using a multimodal analgesia approach for TKA pain management, often involving the combination of adductor canal block (ACB) with periarticular infiltration (PAI) [5, 6]. However, the safety of combining ACB with PAI raises particular concerns due to potential local anesthetic systemic toxicity (LAST), especially with dual doses in bilateral cases. The recommended maximum dose of bupivacaine without epinephrine is 2 mg/kg, not exceeding 175 mg in a single dose or 400 mg within 24 h [7, 8]. When using bupivacaine with epinephrine, the maximum single dose should not exceed 225 mg. However, some studies using higher doses of bupivacaine (200–400 mg) for PAI, with or without ACB, did not report toxicity or adverse events [9–11].

During BTKA procedures at our institution, we typically use total doses of 100 mg bupivacaine for ACB and 200 mg bupivacaine for PAI. Although no cases of LAST have been reported, the use of such high doses of local anesthetics raises concerns about potential toxicity risks. Ropivacaine and levobupivacaine are possible bupivacaine alternatives due to their lower toxicity [8], but they are unfortunately not available in our country. One potential course of action is reducing the PAI bupivacaine dose from 200 mg to a safer 100 mg. However, there are limited data on the efficacy and safety of this reduced amount for simultaneous BTKA.

This study compared the efficacy of two different bupivacaine concentrations. One regimen combined ACB using 100 mg bupivacaine with a PAI containing 200 mg bupivacaine, giving a total of 300 mg bupivacaine. The other regimen combined ACB using 100 mg bupivacaine with PAI containing 100 mg bupivacaine, summing up to 200 mg bupivacaine. The study also assessed plasma bupivacaine concentrations in patients receiving these doses. Other assessments included postoperative pain at rest and during movement, morphine consumption, bupivacaine-related adverse effects, and early postoperative mobilization with walking. The goal was to provide valuable insights into the efficacy and safety of different bupivacaine concentrations for simultaneous BTKA.

## Methods

This prospective, randomized, double-blind, controlled trial was conducted at a large tertiary referral center in Thailand following approval from the Siriraj Institutional Review Board (Si 111/2017) and registration with ClinicalTrials.gov (NCT03249662). The study received support from the Siriraj Research Development Fund (R016033020), Faculty of Medicine Siriraj Hospital, Mahidol University. Written informed consent was obtained from all participants before the study commenced.

### Patients

Eligible participants were scheduled for simultaneous BTKA under spinal anesthesia between September 2017 and March 2020. They were required to be aged 18 years or older and have an American Society of Anesthesiologists physical status ranging from I to III. Exclusion criteria were patient refusal, body weight under 50 kg, renal insufficiency (creatinine clearance less than 60 ml/min), advanced liver disease, uncontrolled cardiovascular diseases (congestive heart failure or unstable angina), preoperative hematocrit value below 35%, allergies to local anesthetics or nonsteroidal anti-inflammatory drugs, inflammation at the venipuncture site, difficult venous access for blood sampling (over two intravenous cannulation attempts), and contraindications to regional anesthesia (coagulopathy or local infection at the injection site).

### Randomization and blinding

**Fig. 1:**
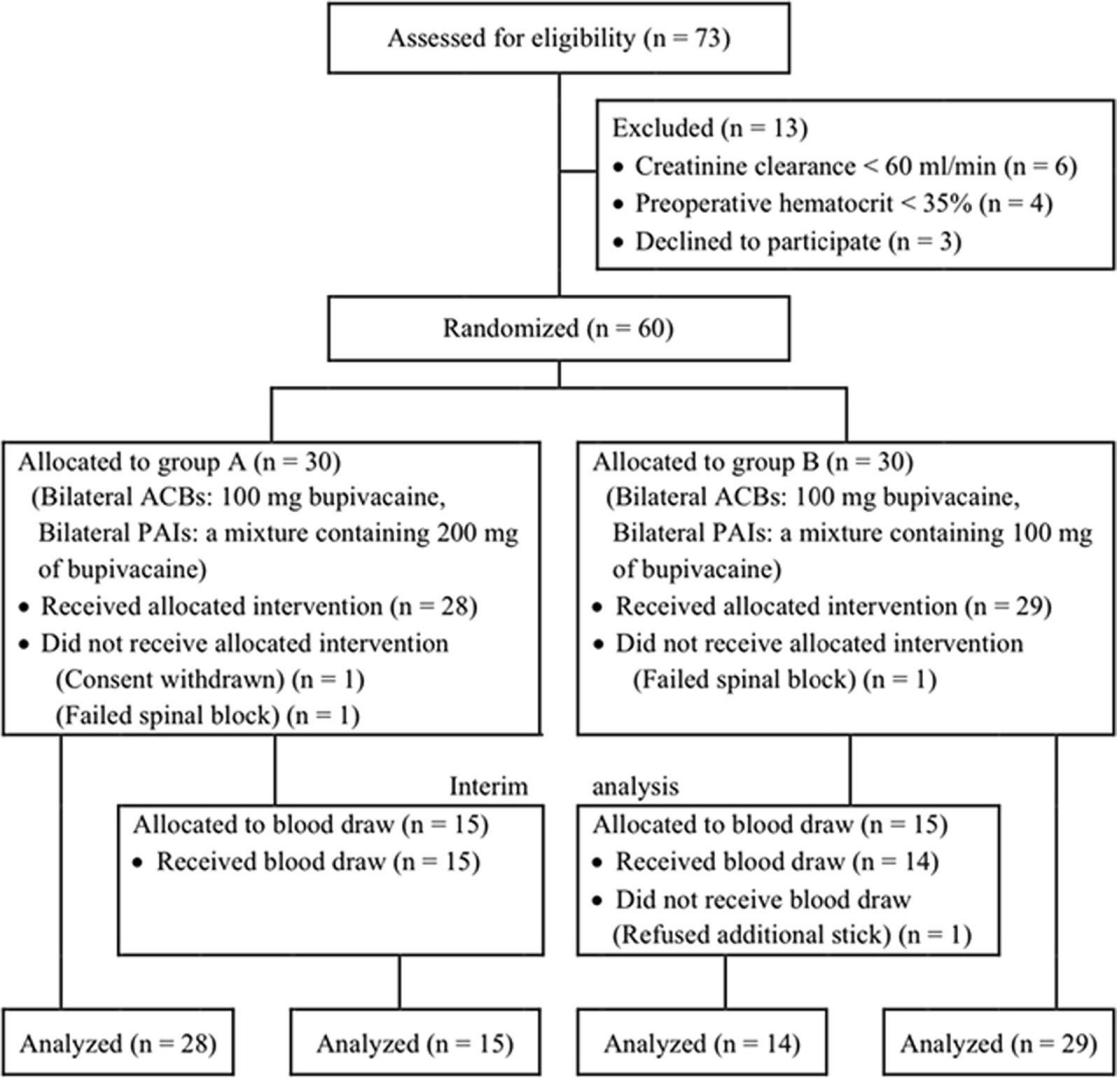
Flow diagram of patient progression. *ACBs* adductor canal blocks, *PAIs* periarticular infiltrations

Randomization of the patients into two groups was performed using computer-generated random numbers. Both groups received PAIs but with different bupivacaine concentrations. In group A, 200 mg bupivacaine (Marcaine 0.5%, AstraZeneca), 30 mg ketorolac (Ketolac, American Taiwan Biopharm), and 400 mcg of 1:1000 epinephrine (5 mcg/mL) were mixed with normal saline to a total volume of 80 mL. In group B, 100 mg bupivacaine, 30 mg ketorolac, and 400 mcg of 1:1000 epinephrine were mixed with normal saline to a total volume of 80 mL. Each patient received 40 mL of their respective solutions per knee. A scrub nurse, unaffiliated with the study, prepared the mixtures. The allocation sequence was concealed until the day of surgery. The surgeons, anesthesiologists, patients, and outcome assessors were blinded to the group allocations. A flow diagram detailing the enrollment and randomization process is presented in Fig. 1.

### Interventions

All participating patients underwent ultrasound-guided ACBs on both thighs, overseen by a senior regional anesthesiologist. The Sonimage HS1 ultrasound machine (Konica Minolta, Japan) with a high-frequency linear array probe (L 11-3) was used for the procedure. The patient was placed in a supine position with the knee slightly flexed, and the leg externally rotated. Using sterile techniques, an ultrasound probe was positioned at the midthigh level to identify the sartorius muscle. The transducer was then moved until the medial border of the sartorius muscle intersected the medial border of the adductor longus muscle. At this level, the femoral artery could be identified underneath the sartorius muscle, and the saphenous nerve, located anterolateral to the artery, was pointed as the target injection site.

Under ultrasound guidance, a 22-gauge 80-mm needle (Ultraplex 360; B. Braun, Melsungen, Germany) was advanced in-plane in a lateral-to-medial direction, aiming to position it close to the saphenous nerve, just lateral to the femoral artery, and deep to the fascia surrounding the posterior part of the sartorius muscle. At this point, a local anesthetic was slowly injected while monitoring its spread around the nerve within the adductor canal. Each thigh was injected with a mixture of 10 mL of 0.5% bupivacaine and 5 mL of 0.9% normal saline, resulting in a total of 100 mg bupivacaine for both ACBs. The successful ACB was assessed by evaluating a cold sensation test along the distribution of the saphenous nerve on the anteromedial side of the thigh. Spinal anesthesia was then given using 2.5–3 mL of hyperbaric or isobaric bupivacaine 0.5% (Marcaine Spinal 0.5%, AstraZeneca). After the anesthesia took effect, the BTKAs were carried out sequentially by one surgical team.

All surgeries were performed by three orthopedic surgeons using the same surgical technique. A medial parapatellar approach was performed in all cases after applying a tourniquet with a pressure of 300 mmHg. Cemented, posterior-stabilized, fixed-bearing knee prostheses (NexGen LPS-Flex; Zimmer Biomet, Warsaw, Indiana) were used for all knees. Bilateral PAIs were performed before closing the wound of each knee using the same technique and local anesthetic solution. Each knee received a total injection of a 40 mL mixture, divided into four areas of 10 mL each: (1) the posterior, posteromedial, and posterolateral capsules; (2) the medial gutter; (3) the lateral gutter; and (4) areas encompassing the quadriceps muscles, retinacular tissue, pes anserinus, and suprapatellar and infrapatellar fat pads [12]. The PAIs consisted of 200 mg bupivacaine for group A, whereas for group B, the injections contained 100 mg bupivacaine. The total amount of bupivacaine administered via the two ACBs and the two PAIs was 300 mg for patients in group A and 200 mg for those in group B.

Patients were closely monitored for signs and symptoms of LAST, such as dizziness, blurred vision, tinnitus, perioral numbness, metallic taste, arrhythmias, agitation, seizures, or cardiac arrest [13]. A treatment plan for LAST was prepared in advance.

Blood samples were periodically drawn during the surgical procedure to determine plasma bupivacaine concentrations. To avoid dilution effects from intravenous fluids or clotting from cuff inflation, a catheter was placed in a large vein separate from the one designated for intravenous fluids and blood pressure monitoring. This catheter was solely for blood sampling purposes. Blood was drawn at eight specific times: 60 min after the bilateral ACBs; before the first PAI; 40 min after the first PAI; and 15, 30, 45, 60, and 90 min after the second PAI. On each occasion, we discarded the initial 3 mL and retained the next 6 mL. After each collection, the catheter was cleansed using 3 mL of a heparin solution (1 unit/mL).

After surgery, each patient followed a standardized multimodal pain management regimen. This regimen consisted of four intravenous doses of parecoxib (40 mg every 12 h), oral Ultracet (a combination of 37.5 mg tramadol with 325 mg acetaminophen, taken twice daily), and oral gabapentin (300 mg at bedtime). Additionally, 2 mg morphine was administered intravenously every 2 h, as needed, for breakthrough pain. To counteract nausea and vomiting, ondansetron (8 mg) was given intravenously every 8 h if needed.

### Outcome measures

The primary outcome assessed in this study was postoperative pain. This was done using a visual analog scale (VAS) ranging from 0 mm (no pain) to 100 mm (worst pain). Pain levels were recorded at 4-h intervals for 48 h after the surgery. The secondary outcomes were the following: plasma bupivacaine concentrations at predetermined time points, average VAS pain scores during rest and movement on the first and second postoperative days, morphine usage within the initial 24 h and the subsequent 24 to 48 h after surgery, occurrences of side effects associated with bupivacaine, and the ability of patients to walk with assistance within the first 2 postoperative days.

### Sample size

The sample size was determined based on the effectiveness of PAIs for pain relief following BTKA. We presumed that a 20-point drop in VAS pain scores would hold clinical significance [14]. Anticipating a standard deviation of 25 for the VAS pain score [14], we estimated that 26 patients per group would be needed to achieve 80% power to detect a difference between the groups at a two-sided significance level of 5%. We recruited 30 patients per group (60 in all) to account for potential dropouts.

### Statistical analysis

The statistical analyses were conducted using PASW Statistics, version 18 (SPSS Inc, Chicago, IL). Categorical data are presented as numbers (percentages), while normally distributed continuous data are presented as means (standard deviations). Non-normally distributed data are presented as medians (interquartile ranges). Comparative analyses between groups were executed using the independent *t* test, Mann–Whitney *U* test, chi-square test, or Fisher’s exact test, depending on the dataset’s nature. Changes in plasma bupivacaine concentrations over time were analyzed using repeated measures analysis of variance. Any *P* value under 0.05 was deemed indicative of statistical significance.

## Results

During the study period, 73 patients who underwent simultaneous BTKA under spinal anesthesia were assessed for eligibility. Of these, 13 patients were excluded before randomization because they did not meet the enrollment criteria or opted not to participate (Fig. 1). The remaining 60 patients were randomly divided into two groups consisting of 30 patients each. However, two patients from group A and one from group B were subsequently excluded due to failed spinal anesthesia and withdrawal of consent. Therefore, 57 patients completed the study, with 28 in group A and 29 in group B. Demographic and perioperative details of both groups are presented in Table 1. No significant differences were observed in age, sex, body mass index, history of diabetes, preoperative hematocrit, creatinine clearance, operative time, or length of hospital stay. Notably, a higher percentage of patients in group B managed to walk with assistance within 2 days after the BTKA procedure than in group A.

**Table 1:** Demographic and perioperative data

Variables	Group A (*n* = 28) (300 mg bupivacaine)	Group B (*n* = 29) (200 mg bupivacaine)	*P*
Age (years)	67.9 (8.5)	67.7 (7.6)	0.925
Female sex	26 (92.9%)	28 (96.6%)	0.611
BMI (kg/m^2^)	27.7 (4.4)	26.8 (4.7)	0.480
Diabetes	6 (21.4%)	4 (13.8%)	0.449
Preoperative Hct (%)	37.5 (3.1)	38.6 (3.0)	0.172
CrCl (mL/min)	85.0 (13.7)	84.7 (14.7)	0.943
Operation time (min)	135.9 (34.1)	120.2 (22.4)	0.061
Morphine consumption (mg)			
0–24 h	6 (3.0–9.8)	6 (3.5–10.5)	0.779
24–48 h	3 (0–8.8)	4 (1.0–6.0)	0.776
Postoperative nausea vomiting	10 (35.7%)	13 (44.8%)	0.483
Walk within 2 days post-TKA	20 (71.4%)	27 (93.1%)	0.041
Length of stay (days)	6.3 (1.2)	6.7 (2.5)	0.405

Data are presented as mean (standard deviation), median (interquartile range), or number (%)

*BMI* body mass index, *Hct* hematocrit, *CrCl* creatinine clearance, *TKA* total knee arthroplasty

**Fig. 2:**
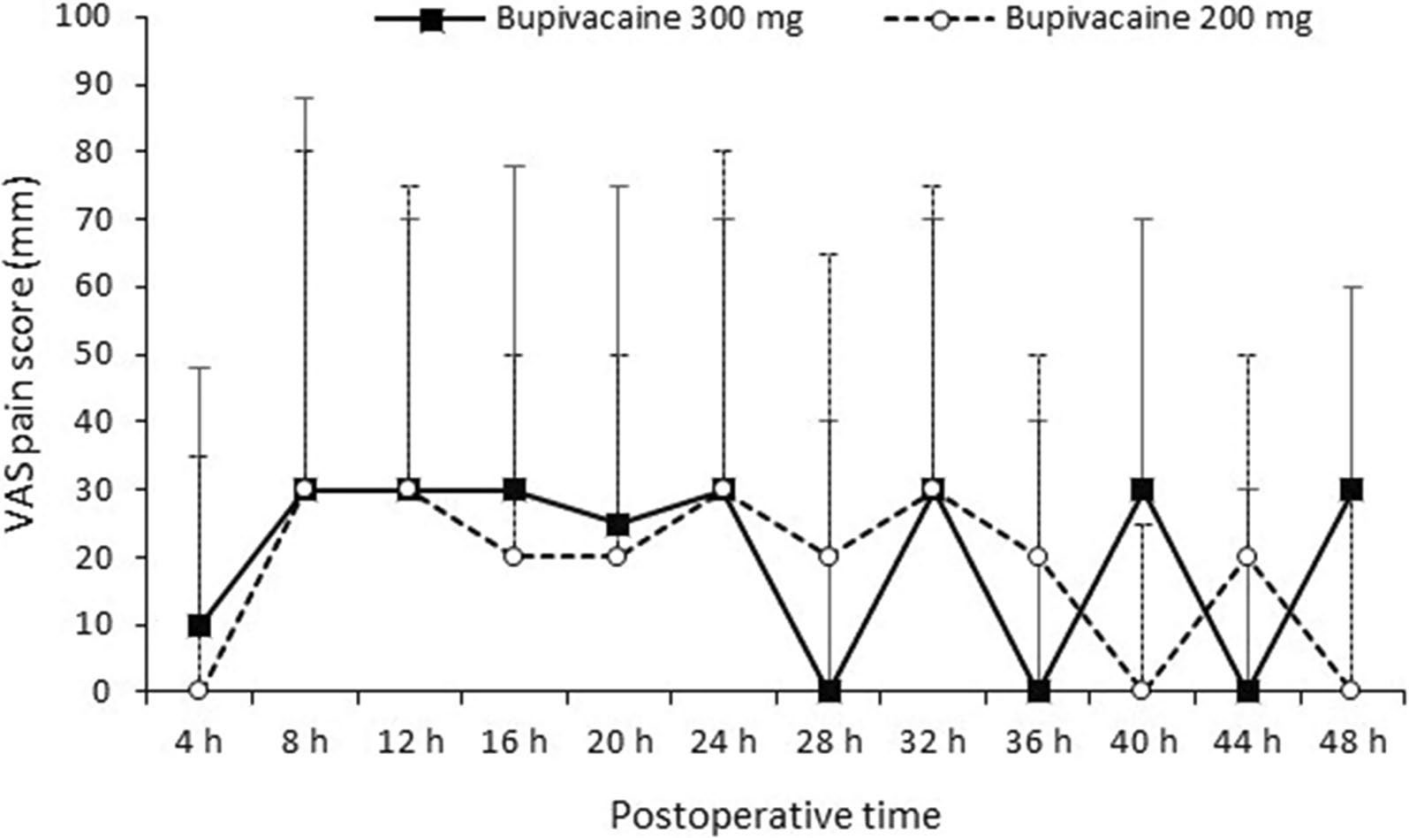
Postoperative visual analog scale (VAS) pain scores (median with interquartile range) after simultaneous bilateral total knee arthroplasty.

**Fig. 3:**
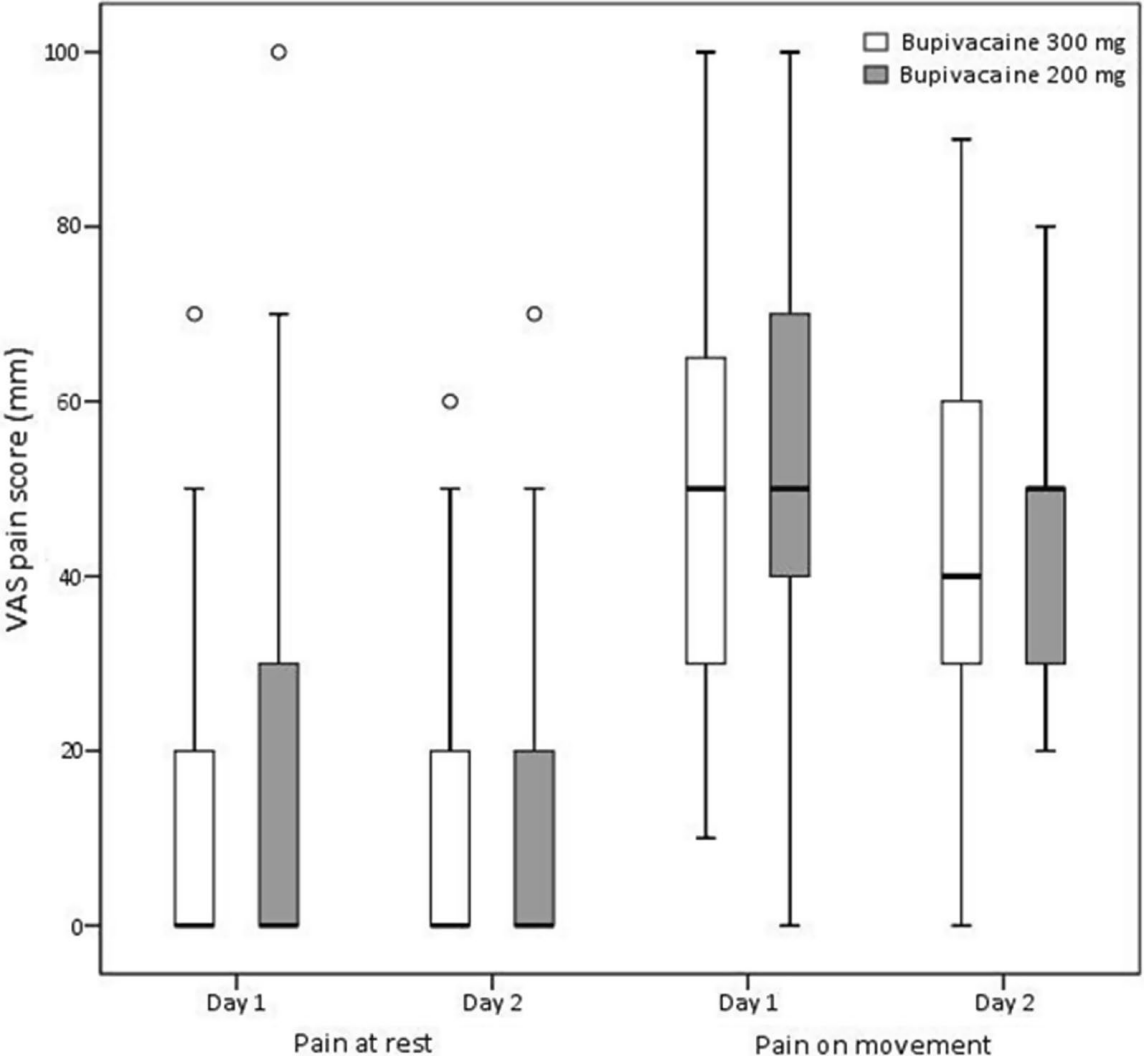
Daily average visual analog scale (VAS) pain scores at rest and during movement after simultaneous bilateral total knee arthroplasty. Scores are presented as medians (central lines), 25th to 75th percentiles (boxes), and 10th to 90th percentiles (whiskers). Outliers are represented by open circles

The median VAS pain scores showed no significant difference between the groups at 4-h intervals from 4 to 48 h postsurgery (Fig. 2). In each group, nine patients reported a VAS score of 70 or higher (severe pain) at least once in the first 24 h after surgery (*P* = 0.928). Four patients (14.3%) in the 300 mg group and two patients (6.9%) in the 200 mg group reported a VAS score of 70 or higher in the subsequent 24 to 48 h after surgery (*P* = 0.423). Additionally, there were no significant differences between the groups in average VAS scores for resting and moving pain (Fig. 3). On postoperative day 1, both groups had a median resting pain score of 0 (*P* = 0.712), which increased to 50 with movement (*P* = 0.765). On postoperative day 2, patients in both groups reported a median resting pain score of 0 (*P* = 0.732). During movement, the 300 mg group had a median pain score of 40, while the 200 mg group had a median pain score of 50 (*P* = 0.750). The consumption of morphine within the first 24 h and 24–48 h was similar in the two groups (Table 1). Three patients (10.7%) in the 300 mg group and two patients (6.9%) in the 200 mg group did not require intravenous morphine for pain relief during the initial 48-h postoperative period. Postoperative nausea and vomiting were not different between the groups (*P* = 0.483).

An interim analysis of plasma bupivacaine concentrations was conducted after collecting blood samples from the first half of the study’s participants. One patient from group B was excluded due to a defective intravenous line and their decision against subsequent venipuncture. Consequently, 29 patients were considered in the analysis: 15 from group A and 14 from group B. No significant differences were observed in the mean plasma bupivacaine levels at any time point between the two groups (Table 2). “Time zero” was defined as the time following the completion of bilateral ACBs.

**Table 2:** Plasma bupivacaine concentrations following bilateral adductor canal blocks combined with periarticular infiltrations

	Group A (*n* = 15) (300 mg bupivacaine)	Group B (*n* = 14) (200 mg bupivacaine)	*P*
*Plasma concentrations (ng/mL)*			
60 min after bilateral ACB	446.5 (222.8)	391.8 (118.4)	0.421
Before PAI of 1st knee	360.4 (193.6)	297.2 (117.4)	0.301
40 min after PAI of 1st knee	369.3 (282.1)	291.1 (133.5)	0.354
15 min after PAI of 2nd knee	378.1 (249.4)	333.5 (143.6)	0.563
30 min after PAI of 2nd knee	355.6 (175.9)	348.8 (171.5)	0.917
45 min after PAI of 2nd knee	510.3 (286.5)	451.0 (164.7)	0.504
60 min after PAI of 2nd knee	483.1 (309.0)	417.8 (162.4)	0.487
90 min after PAI of 2nd knee	499.2 (351.3)	501.6 (154.9)	0.982

Values are presented as mean (SD)

*ACB* adductor canal block, *PAI* periarticular infiltration

Table 3 presents each participant’s maximum plasma concentration (Cmax) and the duration to achieve this peak (Tmax) for each participant. The mean Cmax of bupivacaine in group A was 591.1 (± 347.7) ng/mL, whereas in group B, it was 534.2 (± 138.2) ng/mL. The difference in the two values was nonsignificant (*P* = 0.565). Similarly, the mean Tmax was 2.6 (± 1.4) hours in group A and 3.0 (± 1.2) hours in group B, with no significant variation between the groups (*P* = 0.423).

**Table 3:** Peak plasma concentrations and time to reach peak for each subject

Group A (*n* = 15) (300 mg bupivacaine)			Group B (*n* = 14) (200 mg bupivacaine)		
Subject	Cmax (ng/mL)	Tmax (h)	Subject	Cmax (ng/mL)	Tmax (h)
1a	815.0	1.0	1b	537.7	3.4
2a	337.0	3.3	2b	377.6	1.0
3a	360.9	1.0	3b	491.1	1.0
4a	875.4	2.8	4b	804.1	3.9
5a	481.7	1.0	5b	682.3	3.7
6a	716.3	1.0	6b	636.5	2.9
7a	727.8	3.5	7b	446.3	3.3
8a	1572.9	3.4	8b	324.5	3.4
9a	462.9	4.2	9b	435.2	3.7
10a	350.0	3.4	10b	562.3	3.8
11a	854.4	1.0	11b	712.5	4.5
12a	298.4	4.1	12b	573.2	1.0
13a	425.6	1.0	13b	392.0	3.5
14a	296.1	3.4	14b	503.8	2.4
15a	291.5	4.6			
Mean	591.1	2.6	Mean	534.2	3.0
SD	347.7	1.4	SD	138.2	1.2

*Cmax* maximum plasma concentration, *Tmax* time taken to reach maximum plasma concentration, *SD* standard deviation

The highest individual plasma bupivacaine concentration observed was 1572.9 ng/mL in a patient in group A, occurring at a Tmax of 3.4 h. This measurement was taken 90 min following the second knee’s PAI. In contrast, group B’s highest individual plasma bupivacaine concentration was approximately half as high (804.1 ng/mL). It occurred at a Tmax of 3.9 h and, similarly, 90 min after the second knee’s PAI. Notably, none of the patients in either group reported any symptoms of LAST.

## Discussion

The most important finding of this study is that the combination of ACB (100 mg bupivacaine) and PAI (100 mg bupivacaine) provided comparable pain relief for 48 h postsurgery compared with ACB (100 mg bupivacaine) with PAI (200 mg bupivacaine). No significant differences in plasma bupivacaine concentrations or signs of LAST were observed. Additionally, a higher proportion of patients (93%) receiving ACB and PAI with a total bupivacaine dose of 200 mg could walk with assistance within 2 days after surgery, contrasting with the 71% given a combined total of 300 mg bupivacaine.

Adequate pain relief is crucial for early mobilization and directly influences patient satisfaction following TKA. The PROSPECT Working Group recommends using paracetamol and nonsteroidal anti-inflammatory drugs or cyclo-oxygenase-2-specific inhibitors as the primary analgesics for unilateral primary TKA [6]. Intravenous dexamethasone is also suggested, with opioids reserved for managing breakthrough pain. ACB and PAI are strongly recommended as integral components of a multimodal analgesic approach, offering the advantage of preserving quadriceps muscle strength to facilitate safe early mobilization and rehabilitation. ACB provides analgesia to the anteromedial knee joint by blocking the saphenous nerve and the nerve to the vastus medialis, which traverse the adductor canal of the thigh [15]. On the other hand, PAI provides localized pain relief and reduces inflammation at the surgical site by injecting a combination of local anesthetics, anti-inflammatory drugs, and epinephrine, sometimes including opioids, directly into the tissues surrounding the knee joint [16]. Several meta-analyses have demonstrated a synergistic effect when combining ACB with PAI, resulting in superior pain control in unilateral TKA compared with using either technique alone [17–20].

There are limited available data on pain management in simultaneous BTKA. The consensus conference on BTKA recommended personalized regional anesthesia for each patient [21]. Previous studies have shown the effectiveness of PAI in reducing postoperative pain after BTKA compared with a placebo [22] or no injection [23, 24]. Furthermore, some studies highlighted the advantages of PAI over epidural analgesia [14] or intra-articular injections [16]. However, most studies have focused on comparing PAI to non-PAI for pain control in BTKA. The integration of ACB with PAI and the effects of varied bupivacaine dosages are unexplored.

Our institution’s standard practice for pain management after BTKA involved using 100 mg bupivacaine for ACB and 200 mg for PAI. To minimize the risk of bupivacaine toxicity, we reduced the PAI dose from 200 to 100 mg and conducted a comparative analysis of their efficacy. The results of our study demonstrated that the reduced-dose PAI provided postoperative pain relief and morphine consumption comparable to its higher-dose counterpart.

A study by Koh et al. [25] investigated the efficacy of reduced-dose PAI compared with regular-dose PAI in patients who received continuous epidural analgesia during BTKA. Their findings showed that the reduced-dose PAI resulted in less pain on the night of surgery and similar pain levels on the first postoperative day compared with the standard PAI dose. However, drawing parallels between our study and theirs poses challenges, given differences in methodology, PAI composition, injection technique, and postoperative pain medications. Nevertheless, our study provides evidence that combining ACB with 100 mg bupivacaine and PAI with 100 mg bupivacaine effectively alleviates pain during simultaneous BTKA.

Regarding safety, it is essential to use local anesthetics cautiously to avoid exceeding toxic doses, particularly during high-volume administrations such as PAIs. The reported incidence of LAST ranges from 1.04 to 1.8 per 1000 peripheral nerve blocks for total joint arthroplasty [26, 27]. The most common manifestation of LAST is central nervous system toxicity [8]. Toxicity has been observed at bupivacaine plasma concentrations between 2000 and 4000 ng/mL [28]. Knudsen et al. [29] administered intravascular bupivacaine infusions to healthy volunteers until central nervous system toxicity symptoms emerged. The mean maximum tolerated concentration of bupivacaine in venous plasma was 2100 ng/mL, ranging from 800 to 4500 ng/mL. A similar trial by Bardsley et al. [30] yielded comparable results, with a mean Cmax of bupivacaine of 2250 ng/mL.

Our study measured bupivacaine plasma concentrations in patients who underwent simultaneous BTKA and received either 300 or 200 mg bupivacaine for ACB plus PAI. For those receiving 300 mg, the mean Cmax of bupivacaine was 591.1 ng/mL. Similarly, for patients receiving 200 mg, the mean Cmax of bupivacaine was 534.2 ng/mL. The Cmax levels of bupivacaine in both dosage groups showed no significant differences and remained below the reported toxic threshold for central nervous system toxicity. However, there has been a reported case of a young and healthy patient experiencing convulsions after receiving an intravenous bupivacaine infusion at a rate of 2 mg/min, with a plasma concentration of bupivacaine at 1100 ng/mL at the time of the incident [31].

One patient in our 300 mg group had the highest individual Cmax value of bupivacaine (1572.9 ng/mL), while no patients in the 200 mg group had a Cmax value of bupivacaine exceeding 1000 ng/mL. Although no signs or symptoms of systemic bupivacaine toxicity were observed in either group, the safety margin for bupivacaine appeared to be wider in the 200 mg group than in the 300 mg group. Therefore, 200 mg bupivacaine in ACB plus PAI may be suitable for simultaneous BTKA. Additionally, the longest individual Tmax in group A was 4.6 h, whereas in group B, it was 4.5 h. To ensure safety, it is advisable to administer the lowest effective dose of the local anesthetic and closely monitor the patient for a minimum of 4.6 h after completing ACB.

Walking shortly after TKA enhances recovery and joint mobility but is often hindered by postoperative pain. Combining ACB and PAI may alleviate walking pain [18] and increase walking distance [19, 20] compared with using PAI alone. In our study, more patients receiving a total 200 mg bupivacaine dose for ACB plus PAI could walk with assistance within 2 days after simultaneous BTKA than those with a 300 mg dose, while pain during movement did not differ between the two groups. The reason for the difference in walking ability is unclear, requiring further investigation.

One potential rationale behind these outcomes could be the differential impacts of local anesthetic concentrations on quadriceps muscle strength. Additionally, the analgesic efficacy of local anesthetics depends on the concentration of the remaining drug at the injection site and its absorption rate into the circulatory system [28]. Individual variability may also contribute to the observed differences in drug effects. While the precise reason for the observed variance in early ambulation remains speculative, these findings have potential clinical relevance. A more in-depth investigation is warranted for a conclusive understanding.

This study had several limitations. First, the power of secondary outcomes (plasma) was underpowered, involving a small number of patients. Second, the ACB technique could also affect functional recovery, potentially introducing heterogeneity due to its effect on the quadriceps. Third, the ACBs were performed by multiple trainees under the supervision of regional anesthesiologists, which may have introduced variations in the techniques used. Fourth, our focus was solely on the cumulative plasma concentrations of bupivacaine. The toxicity of local anesthetics traditionally correlates more with the free (unbound) plasma concentrations as opposed to the overall (both bound and free) concentrations [28]. While the total bupivacaine plasma concentration provides valuable information, the free plasma concentration is a more accurate gauge of potentially toxic levels of bupivacaine. Fifth, in group A, plasma bupivacaine levels decreased after reaching their peak concentration, whereas in group B, they peaked 90 min after the second knee injection. It remains uncertain whether the plasma bupivacaine levels increased or decreased after the last blood collection. This highlights the need to continue monitoring plasma bupivacaine levels over a sufficient duration and to be alert for LAST symptoms. Finally, our study’s assessment of post-TKA functional recovery was solely based on the capability to walk with assistance within 48 h. Broader measures, such as pain during walking, distance traversed, or joint flexibility, might offer enriched insights.

## Conclusions

This randomized controlled trial demonstrated that combining ACB and PAI with a total dose of 200 mg bupivacaine provided similar pain relief and a wider safety margin for simultaneous bilateral TKA than a total dose of 300 mg bupivacaine. Additionally, a higher proportion of patients who received the 200 mg dose could walk with assistance within 2 days after surgery. Therefore, ACB plus PAI with 200 mg bupivacaine appears to be a suitable choice for simultaneous bilateral TKA. However, further investigation is needed to determine the effectiveness of this dose in promoting early knee function recovery.

## Data Availability

The datasets generated and/or analyzed during the present study are not publicly available due to internal institutional restrictions, but they are available from the corresponding author upon reasonable request and with the permission of the institution where the data were generated.

## Abbreviations

ACB: Adductor canal block
BTKA: Bilateral total knee arthroplasty
Cmax: Maximum plasma concentration
LAST: Local anesthetic systemic toxicity
PAI: Periarticular infiltration
TKA: Total knee arthroplasty
Tmax: Time taken to reach maximum plasma concentration
VAS: Visual analog scale
